# Remineralization Efficacy of an Amelogenin-Based Synthetic Peptide on Carious Lesions

**DOI:** 10.3389/fphys.2018.00842

**Published:** 2018-07-05

**Authors:** Jinpu Chu, Xiaofang Feng, Huijing Guo, Tieting Zhang, Hualei Zhao, Qun Zhang

**Affiliations:** ^1^The First Affiliated Hospital of Zhengzhou University, Zhengzhou, China; ^2^College of Stomatology, Zhengzhou University, Zhengzhou, China

**Keywords:** amelogenin-based synthetic peptide, early enamel lesion, biomimetic, remineralization, amorphous calcium phosphate

## Abstract

**Objective:** The aim of this study was to evaluate the remineralization efficacy of an amelogenin-based peptide on initial enamel carious lesions *in vitro*. Furthermore, we attempted to provide insights into the possible mechanism of the remineralization, including determining the calcium-binding properties of the peptide and its effects on calcium phosphate mineralization.

**Methods:** The peptide comprising the N-terminus and the C-terminus of porcine amelogenin was synthesized by Synpeptide Co., Ltd. Fifty specimens were randomly assigned to five immersing treatment groups for 12 days: remineralizing medium only; 12.5 μg/mL peptide + remineralizing medium; 25 μg/mL peptide + remineralizing medium; 50 μg/mL peptide + remineralizing medium; fluoride + remineralizing medium. After immersion, mean mineral loss before and after remineralization of each specimen was determined using micro-CT. Mean mineral gain after remineralization was calculated. Calcium binding properties were measured by Isothermal titration calorimetry (ITC). TEM and Fourier transform-infrared were used to determine the effects of the peptide on calcium phosphate mineralization.

**Results:** A significant decrease in mineral loss after remineralization process in all groups was observed (*p* < 0.05). Treatment in remineralizing medium resulted in the lowest mineral gain while the fluoridated treatment exhibited the highest mineral gain among all groups. Inclusion of synthetic peptide in the remineralizing medium exhibited a higher mineral gain and the gain of 50 μg/mL group was greater than that of the 25 μg/mL group. No significant difference in mineral gain was observed between the remineralizing medium only group and the 12.5 μg/mL peptide group (*p* > 0.05). ITC values showed that the Ca^2+^-binding affinity of the peptide is about 9.914 × 10^4^M^−1^. Furthermore, the peptide was found to inhibit calcium phosphate precipitation and stabilize amorphous calcium phosphate formation for more than 2 h and finally transform into ordered hydroxyapatite crystals.

**Conclusion:** Specific concentrations of the amelogenin-based synthetic peptide promoted *in vitro* remineralization, with higher concentrations exhibiting significantly greater remineralization. This study presented evidence suggesting that the peptide may act as a Ca^2+^carrier as well as a regulating factor. When the stabilizing calcium and phosphorus ions bind with the peptide they become biologically available for the remineralization of deeper carious lesions, while also regulated by the peptide to transform into ordered hydroxyapatite crystals.

## Introduction

Dental enamel is a unique mineralized substance that is comprised mainly of hydroxyapatite (92–94%). Because of the lack of cellular repair mechanisms, the events surrounding the development and reversal of caries are dependent upon physicochemical events at the tooth-pellicle/plaque interface (Hicks et al., 2004). This remains an important health issue worldwide and occurs when the dynamic demineralization–remineralization balance in enamel is altered by acid-producing bacteria during fermentation of dietary sugars (Ehrlich et al., 2009). This is a dynamic process which can be diagnosed and cured during the early stage. Therefore, the focus of modern dentistry has recently shifted to developing methods for early detection of carious lesions and non-invasive treatment options such as remineralization, in order to prevent disease progression and improve esthetics, strength, and function. Remineralization is believed to occur by the transfer of mineral ions, such as calcium and phosphate, from saliva, plaque fluid, or an external source to the surface of solid-state mineral, therefore promoting ion deposition into crystal voids caused by demineralization (Elkassas and Arafa, 2014). Various modalities of fluoride (Brighenti et al., 2006; Majithia et al., 2016), casein phosphopeptides and amorphous calcium phosphate (ACP) (Poggio et al., 2013; Gupta et al., 2016), sodium trimetaphosphate (Takeshita et al., 2016), and some natural products (Zhang et al., 2015; Cardoso et al., 2016) have been reported to promote remineralization of incipient demineralized lesions. In particular, fluoride dentifrices are widely used for caries prevention and reversal. However, the lack in ability to guide the formation of mineral crystals makes it difficult for fluoride to form ordered mineral crystals on the caries lesions under physiological conditions (Fan et al., 2009). Therefore, there is a need for an alternative approach that can safely and effectively promote organized mineral deposition onto dental tissues.

With better understanding of the biomineralization progress, using biomimetically synthesized enamel-like hydroxyapatite to rebuild tooth enamel and preserve tooth structure has become a popular interest among research groups (Fan et al., 2009; Le Norcy et al., 2011a). Biomimetic remineralization mimics enamel biomineralization, where the organic matrix mediates the formation of hydroxyapatite crystals through protein/inorganic material interactions. Enamel matrix proteins (EMPs), which are secreted by ameloblast during the secretory stage of amelogenesis, are believed to play an important role in the control of crystal growth, including crystal size, shape, and organization, during enamel biomineralization (Zeichner-David, 2011). In particular, amelogenin, which makes up more than 90% of the enamel matrix, is a relatively hydrophobic protein composed of three domains: a N-terminal tyrosine-rich domain, which contains a phosphate group on serine 16; a charged hydrophilic carboxyl terminus; and a large central hydrophobic domain. Amelogenin is believed to self-assemble into supramolecular structures (Fincham et al., 1995) that plays a direct role in initiating nucleation (Tarasevich et al., 2007), controlling crystal growth (Beniash et al., 2005), and affecting the spacing of crystallites (Moradian-Oldak et al., 1998). Ultimately, amelogenin can guide the formation of highly anisotropic and ordered apatitic crystals during enamel development. Numerous *in vitro* experimental approaches have explored the role of amelogenin in enamel mimetic biomineralization: amelogenin promotes the oriented bundle formation of needle-like fluoridated hydroxyapatite (Ruan et al., 2014), and can form an enamel-like layer on an etched enamel surface (Fan et al., 2009) in a dose-dependent manner. However, the role of amelogenin in biomimetic remineralization of early enamel lesions has not yet been fully studied.

**FIGURE 1:**
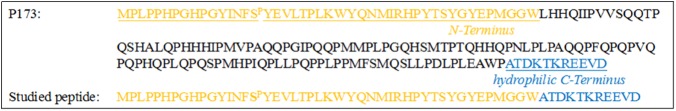
Amino acid sequence of the full-length native porcine amelogenin P173 and synthetic peptide studied here. Note that the peptide is composed exclusively of the N- and hydrophilic C-terminal domains of the full-length molecule. Both the native protein and synthetic peptide were phosphorylated at S-16.

Previous reports have found that the N- and C-terminal domains of amelogenin have abundant charged amino acid residues (Lu et al., 2013). The N-terminus contains phosphorylated serine, which plays an important role in stabilizing ACP and preventing unwanted mineralization (Le Norcy et al., 2011a,b; Margolis et al., 2014). The hydrophilic C-terminal is essential for the arrangement of the crystals into parallel arrays (Beniash et al., 2005; Kwak et al., 2009). Moreover, there is high homology of the N- and C-terminal domains across different species (Fincham et al., 1999), which suggest that these regions play specific functional roles during matrix-mediated enamel biomineralization. Here, we designed and synthesized a novel amelogenin-based peptide, only composed of the functionality amino acid residues of the N-terminus (contains a phosphate group on serine 16) and the hydrophilic C-terminus of porcine amelogenin (**Figure 1**). We hypothesize that this peptide may promote *in vitro* biomimetic remineralization of early enamel lesions. In this investigation we tested the remineralization efficacy of the synthesized peptide on an *in vitro* model of bovine enamel caries, and quantified remineralization by micro-CT. Moreover, we examined possible remineralization mechanisms of our peptide.

## Materials and Methods

### Enamel Specimen Preparation and Peptide Synthesis

**FIGURE 2:**
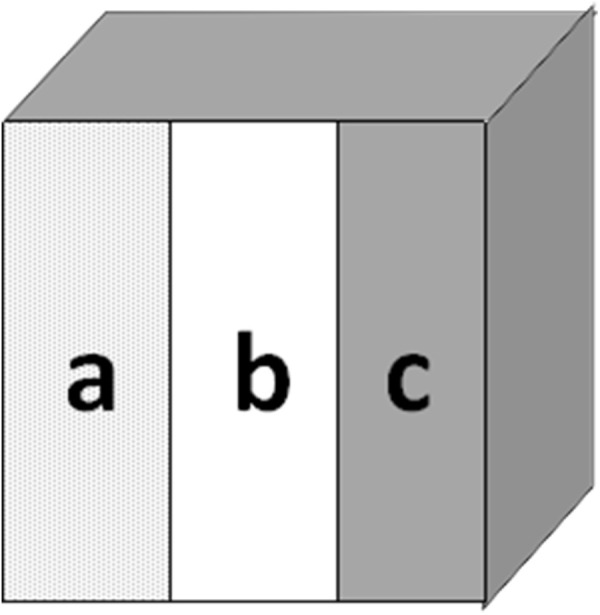
Bovine specimens from the buccal surface. Window **(a)** served as baseline enamel, window **(b)** served as test window, and window **(c)** served as sound caries.

Extracted bovine teeth were collected according to guidelines approved by the Ethics Committee of the First Affiliated Hospital of Zhengzhou University. Enamel disks (3 mm × 3 mm × 2 mm) were obtained from the buccal surfaces of bovine incisors, and were cleaned and inspected for observable cracks, white spot lesions, or enamel malformation under a stereoscopic microscope. The superficial enamel surfaces of the disks were then serially polished with water-cooled silicon-carbide disks (320, 600, 800, 1200, 1500, 2000, and 2500 grade of Al_2_O_3_ paper; Buehler Ltd.), and uncontaminated surfaces were obtained by removing the outermost ∼150 μm and cleaning in an ultrasonic device with de-ionized water for 10 min. For each specimen, we divided each 3 mm × 3 mm buccal window into three windows of 3 mm × 1 mm (**a**, **b**, and **c**, **Figure 2**). All surfaces of each specimen were coated with acid-resistant nail varnish, except for windows **a** and **b**. Window **a** served as the baseline caries, window **b** served as the test window, and window **c** served as the intact enamel reference (Songsiripradubboon et al., 2014).

The peptide, comprising the first 45 amino acid residues of the N-terminus (which contains a phosphate group on serine 16) and the last 11 residues of the C-terminus of porcine amelogenin, was synthesized by Synpeptide Co., Ltd., (Shanghai, China) using standard solid-phase peptide synthesis, and purified and identified using HPLC and ESI-MS. Lyophilized peptide was dissolved in Millipore-purified water at 5–10 mg/mL and stored at 4°C. Peptide stock solutions were centrifuged (10,900 × *g*, 4°C, 20 min) just before use.

### Carious Lesion Formation

To produce artificial carious lesions, 50 enamel specimens were exposed to 3 mL of demineralization solution (DS) per specimen at 37°C for 3 day under continuous magnetic stirring at 100 RPM. DS was prepared as previously reported (Ten and Duijsters, 1983). The pH was adjusted to 4.5 using a KOH (Sigma, St. Louis, MO, United States) solution. The treatment leads to the formation of subsurface lesions ∼100 μm in depth. Each enamel specimen was rinsed carefully with sufficiently distilled deionized water and air-dried after the demineralization. Window **a** was coated with acid-resistant nail varnish to act as a baseline control.

### Remineralization Testing

The 50 bovine specimens were randomly assigned to five different remineralizing treatments (10 specimen/group): **A**, remineralizing medium only; **B**, 12.5 μg/mL peptide + remineralizing medium; **C**, 25 μg/mL peptide + remineralizing medium; **D**, 50 μg/mL peptide + remineralizing medium; **E**, fluoride + remineralizing medium. All treatments were performed at 37°C. The remineralizing medium was prepared as previously reported (Ten and Duijsters, 1983). It consisted of 1.5 mM CaCl_2_, 0.9 mM KH_2_PO_4_, 130 mM KCl, 20 mM HEPES, and 5 mM NaN_3_, where the pH was adjusted to 7.4 with KOH. Fluoridated remineralizing medium was prepared by adding NaF to a final fluoride concentration of 2 ppm just before treatment. We sequentially added aliquots of calcium and pH-adjusted phosphate solutions (pH 11.3 ± 0.1) to peptide stock solutions to yield final concentrations of 1.5 mM CaCl_2_, 0.9 mM KH_2_PO_4_. All reagents were purchased from Sigma-Aldrich (Sigma, St. Louis, MO, United States).

Blocks of each group were suspended in 15 mL (1.5 mL per specimen) of corresponding remineralizing medium at 37°C for 12 days under continuous magnetic stirring. Remineralizing media was replenished every 2 days, and the pH of the solutions was checked and adjusted if necessary. After the remineralization treatment the enamel samples were washed with sufficiently distilled deionized water and air-dried.

### Micro-CT Scan

After the remineralization treatment, the mineral density and profiles of each specimen were determined with an Inveon micro-CT system (Siemens AG, Germany). The micro-CT X-ray source was set at an operating voltage of 80 kV and 500 μA, providing a 10-μm isotropic voxel resolution (Zhang et al., 2016). A mineral density calibration curve was generated by scanning three hydroxyapatite disks (50, 250, and 750 mg/mL) as reference phantoms (Songsiripradubboon et al., 2014). The system was calibrated before each scan.

Three-dimensional images of the enamel specimen were reconstructed for the micro-CT analyses. An Inveon MM Platform (model 5001) was used for visualization and analysis of the volumetric data. A linear calibration curve based on the gray values were obtained from the mineral reference phantoms (linear regression, *R*^2^ > 0.9966), which was utilized to convert CT values into mineral density values (MD) (mg/mL). The assessment was performed at the center of the baseline, test, and sound windows of each specimen, respectively, in a volume of interest (VOI) of 300 μm × 200 μm × 200 μm. Mean MD were calculated at each 10-μm depth in the VOI. By plotting the MD against the depth, the MD profile of the baseline, test, and sound windows in each specimen were obtained. The percent remineralization (%R) of each specimen was calculated using the following formula:

$$\%R=\frac{\left(c-a)-(c-b\right)}{c-a}\times 100$$

Where *a* is the baseline base; *b* is the test base; *c* is the sound base; (*c*−*a*) is mineral loss after demineralization; (*c*−*b*) is mineral loss after remineralization; and (*c*−*a*)−(*c*−*b*) is relative mineral gain due to remineralization.

### Calcium-Binding Test

Isothermal titration calorimetry (ITC) was used to analyze the thermodynamics of Ca^2+^ binding to the synthetic peptide. The ITC measurement was performed in the standard volume nanoITC (TA Instruments^®^) and carried out at 37°C while stirring at 400 rpm. The reference cell was filled with water. The peptide solution was prepared in 10 mM HEPES (pH = 7.4) 1.25 mM Ca^2+^ was injected into 0.12 mM synthetic peptide solution. The Ca^2+^ was titrated into peptide solution via 24 individual injections (10 μL for each) at the interval of 120 s. Blank experiment was measured by titrating Ca^2+^ into 10 mM HEPES under the same conditions. The data were acquired using ITCRun data acquisition software.

### Spontaneous Mineralization Testing

Peptide solution of 50 μg/mL was prepared as described above. Stock solutions of Ca^2+^ (30 mM) and Pi (3 mM) were prepared by reagent grade CaCl_2_.2H_2_O (Sigma, St. Louis, MO, United States, >99.0% pure) and KH_2_PO_4_ (Sigma, St. Louis, MO, United States, >99.0% pure). The KH_2_PO_4_ solution was adjusted to pH 10∼11 at 25°C, using small quantities of KOH. All these solutions were filtered (0.22-μm Isopore filters, Millipore, Billerica, MA, United States) before use. Aliquots of stock solutions of CaCl_2_.2H_2_O and pH-adjusted KH_2_PO_4_ were added to the cold synthetic peptide solution to give a final concentration of 2.5 mM Ca^2+^, 1.5 mM Pi, and 50 μg/mL peptide (final volume 0.06 mL), as previously reported (Kwak et al., 2009). The reaction solutions would have an initial pH ∼7.4 at 37°C by mixing all solutions. The samples were then transferred into a thermostatic water bath adjusted to 37°C. A duplicate sample was also prepared to monitor changes in pH as a function of time by a micro-combination pH electrode (FE20k, Mettler toledo, CH). Subsequently, 5-μL aliquots were taken for TEM analyses at 15 min, 45 min, 2 h, and 24 h from the beginning of the experiments. These aliquots were placed on Ultra-thin carbon support film (Yasheng Electronics Technology Co., LTD., United Kingdom) for 1 min, and then blotted vertically using filter paper, and finally air dried. The grids were examined in both bright-field and selected area electron diffraction (SAED) modes using a JEM-2100 TEM microscope at 100 kV and captured using an AMT CCD camera (AMT, Danvers, MA, United States), as previously described. These images were analyzed using DigitalMicrograph 3.7. Finally, the 24-h sample was concentrated to confirm the SAED findings, using Fourier transform-infrared (FT-IR). The sample was placed on a KBr IR Card (International Crystal Labs, Garfield, NJ, United States), and then the spectra (4000 to 450 cm^−1^) of these samples were recorded using FT-IR (Nicolet 6700, United States). Control experiments were carried out in similar fashion but without the addition of peptide.

### Data Analysis

Remineralization data was analyzed with SPSS 21.0 (IBM, Chicago, IL, United States) using α = 0.05. Paired *t*-test was used to assess the changes in mineral loss values before and after remineralization cycles within each group. The %R and mineral gain among groups were analyzed by one-way ANOVA followed by Newman–Keuls test for multiple comparisons. Calcium-binding results were analyzed with Nano Analyze Software, and the “independent” model was used for data fitting.

## Results

### Effects on the Remineralization of Demineralized Bovine Enamel

**FIGURE 3:**
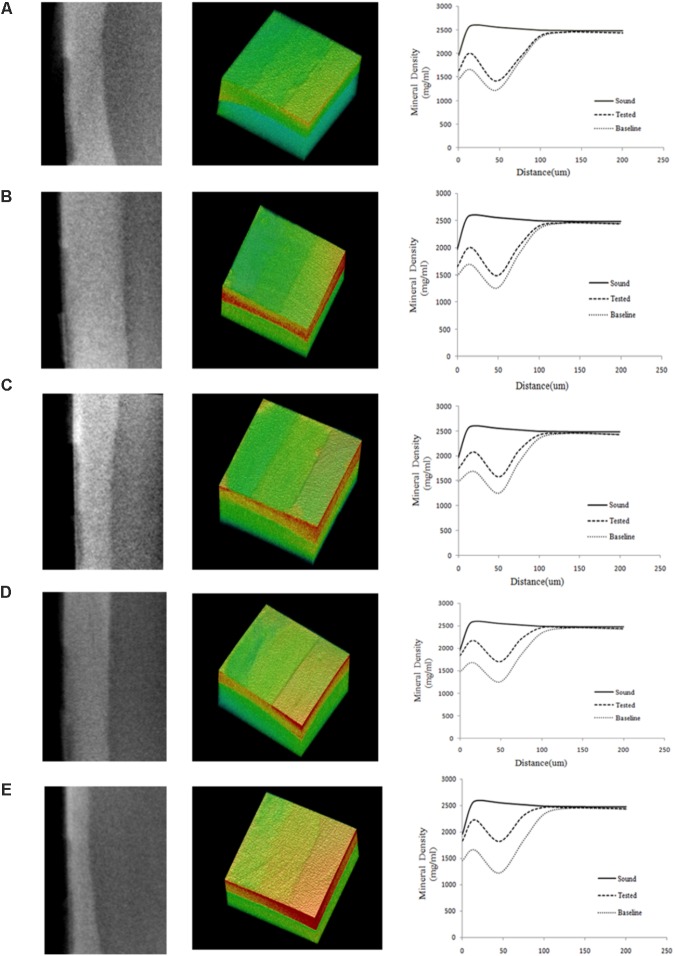
Typical 2D and 3D micro-CT images of all the experimental groups. **(A)** Being remineralizing medium only; **(B)** Being 12.5 μg/mL peptide; **(C)** Being 25 μg/mL peptide; **(D)** Being 50 μg/mL; **(E)** Being fluoridated remineralizing medium. More red means higher mineral density, more green means lower mineral density. The graphs show the mean mineral density profiles of the test, sound, and baseline caries windows, with the distance (*x*-axis, mm) and mineral density (*y*-axis, mg/mL). Group A showed increased mineral density at 0–50 μm and little changes at 50–100 μm from the surface of specimen. The other media (group B, C, D, and E) had obvious effects not only on the superficial layer but also on the deeper layer of the lesion. Each for measurement of mineral density was set through the enamel surface in a volume of interest (VOI) of 300 μm × 200 μm × 200 μm at the center of the window.

**Figure 3** shows typical 2D and 3D micro-CT images of the bovine samples in the different experimental groups. Each specimen contained the baseline, test, and sound windows, and representative profiles of mineral density versus depth from the surface of specimens are also shown. We found that the mineral density converged at a depth of approximately 100 μm for all treatment groups. The remineralizing only medium (group A) showed increased mineral density at 0–50 μm and little changes at 50–100 μm from the surface of the specimen. The other media (group B, C, D, and E) had obvious effects not only on the superficial layer but also on the deeper layer of the lesion.

**FIGURE 4:**
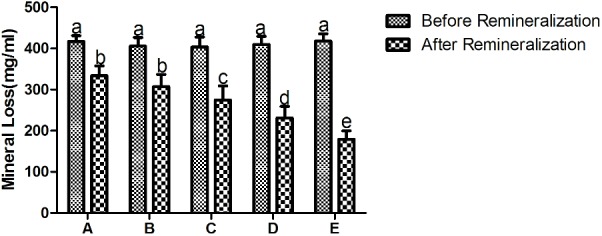
The mineral loss of different treatment groups before and after remineralization. **A** being remineralizing medium only; **B** being 12.5 μg/mL peptide; **C** being 25 μg/mL peptide; **D** being 50 μg/mL; **E** being fluoridated remineralizing medium. Same letters indicate no significent difference in the same chart. It shows a significant decrease in mineral loss of all groups after treatment.

As shown in **Figure 4**, the mineral loss of all the experimental groups is significant after demineralization treatment. Student’s paired *t*-test was used to compare mean mineral loss before and after remineralization cycles. There was a significant decrease in mineral loss of all groups after exposure to the remineralizing medium, and even the remineralization group only (treatment A) exhibited statistically significant difference in mineral loss after remineralization treatment.

**Table 1** illustrates the mean mineral gain from the MD profiles of each group after remineralization and the percent remineralization of each group. The data is described using mean ± standard deviation. The fluoridated remineralizing medium group (treatment E) exhibited a higher mineral gain after treatment compared to the other groups. Treatment in remineralizing medium only (treatment A) resulted in the lowest mineral gain among all groups. The addition of 12.5 μg/mL peptide to the remineralizing medium yielded obvious mineral gain, but no significant difference was observed when compared to the remineralizing medium only group. Peptide concentrations of 25 and 50 μg/mL both obtained relatively higher mineral gains compared to the remineralizing medium only group and the 12.5 μg/mL peptide group. The effect of 50 μg/mL peptide group was greater than that of the 25 μg/mL group. The analyzed results of mean %R was consistent with the results of mean mineral gain. Twelve point five microgram per milliliter peptide, 25 μg/mL peptide, 50 μg/mL peptide, and fluoridated remineralization solution resulted in a 22.51, 62.01, 119.75, and 187.64 increase in %R, respectively, compared with the remineralization solution.

**Table 1:** Remineralization of initial carious lesions after each experimental phase.

	Mean mineral gain (mg HAP.cm–3 μm)	% Remineralization	% Remineralization compared to the remineralization medium only
The remineralizing medium only	82.62 ± 11.90^a^	19.90 ± 3.36^e^	0
12.5 μg/mL peptide	98.38 ± 13.85^a^	24.38 ± 4.20^e^	22.51
25 μg/mL peptide	129.26 ± 14.92^b^	32.24 ± 5.15^f^	62.01
50 μg/mL peptide	178.76 ± 18.27^c^	43.73 ± 5.01^g^	119.75
Fluoridated remineralizing medium	239.09 ± 18.09^d^	57.24 ± 4.28^h^	187.64

*Same letters indicate no significant difference in the same column.*

### Effects on Ca^2+^-Binding

**FIGURE 5:**
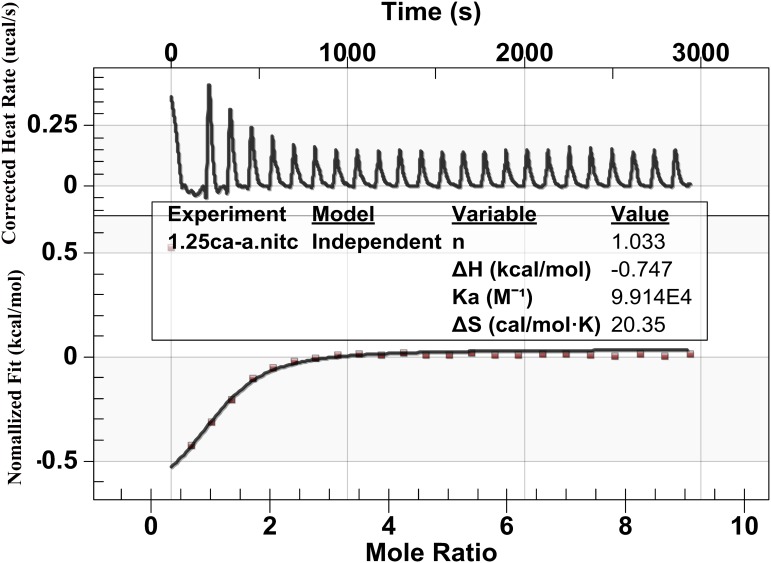
Binding analysis of Ca^2+^ to peptide using ITC: The top panel shows the raw titration data measured in μcal/s. Each peak corresponds to a single injection of Ca^2+^ into the peptide, and the bottom graph represents integrated heat data after corrections of dilution heat against molar ratio of Ca^2+^/peptide fitted to the “independent binding sit.” It shows that the binding constant Ka of Ca^2+^/peptide is about 9.914 × 10^4^ M^−1^ and the stoichiometry of binding (*n*) is about 1 mol of Ca^2+^ per mole of peptide.

**Figure 5** shows the heat generation of each injection (ucal/s) between calcium ions and the peptide against time(s). Upward peaks indicate an exothermic reaction. The heat of each injection returned rapidly to the baseline, indicating that the Ca^2+^ and peptide reacted rapidly. Each successive peak after the eighth presented similar magnitude, indicating binding saturation. The bottom diagram illustrates the effective binding isotherms of Ca^2+^/peptide interactions after correction for background effects. The analysis of the thermodynamic values showed that the binding constant Ka of Ca^2+^/peptide was found to be up to 9.914 × 10^4^ M^−1^, suggesting that binding Ca^2+^ to the peptide may be easier and more stable. The enthalpy change (ΔH) of binding is −0.747 kcal/mol and entropy change (ΔS) is 20.35 cal/mol k. The stoichiometry of binding (*n*) is about 1 mol of Ca^2+^ per mole of peptide.

### Effects on Spontaneous Mineralization

**FIGURE 6:**
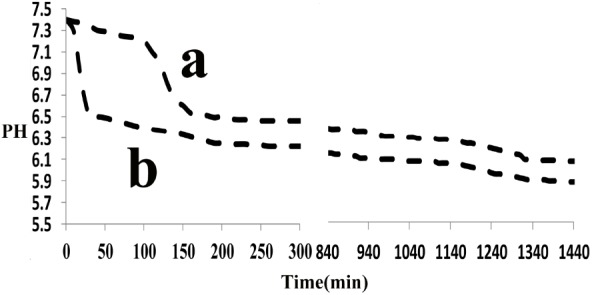
Changes in pH observed during Spontaneous mineralization experiment as a function of time: in the absence (control, line b), in the presence of peptide (line a), monitored over a 24-h period. In the absence of peptide, a rapid decrease in pH was observed after 15 min but it was observed after 2 h in the presence of peptide. Similar results were obtained for multiple repeats (*n* = 3).

**FIGURE 7:**
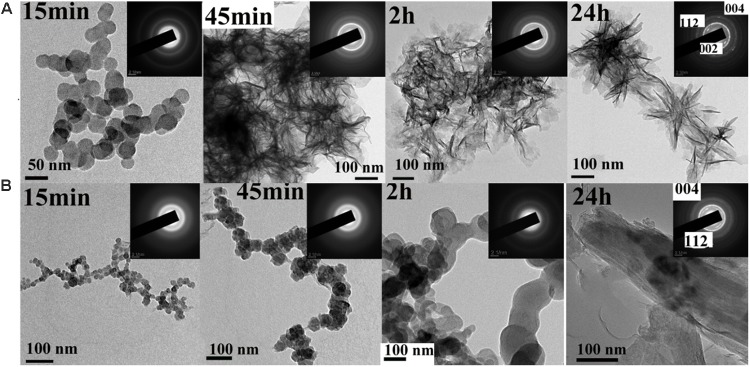
TEM and SAED (insets) analyses of mineral phases formed during spontaneous mineralization experiments in the absence (**A**, control) and presence of peptide **(B)** examined at selected times (15 min, 45 min, 2 h and 24 h). As shown at 15 min, ACP was initially formed in the absence and presence of peptide. ACP was also form at 45 min even at 2 h in the present of peptide, whereas crystals were observed at this time in the absence of peptide. After 24 h, randomly arranged plate-like apatitic crystals were found in the absence of peptide, whereas aligned bundles of ribbon-like apatitic crystals were formed in the presence of peptide.

**FIGURE 8:**
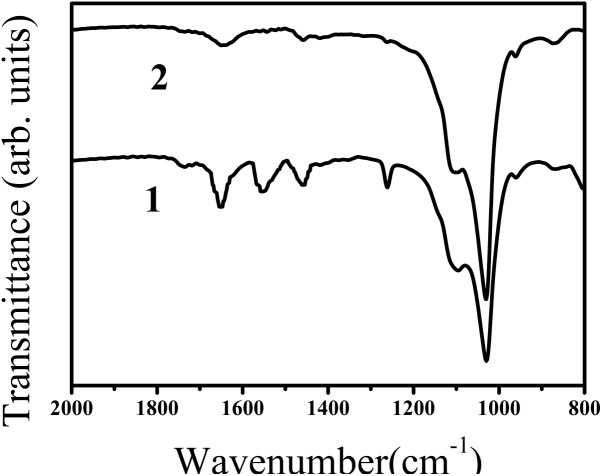
FT-IR spectra of calcium phosphate mineral phases formed after 24 h in the absence (curve 1, control) and presence of peptide (curve 2). In both of the curves, ν3 of PO_4_^3-^ peaks (1100 and 1030 cm^−1^, curve 1) and (1095 and 1029 cm^−1^, curve 2) were observed suggesting the formation of hydroxyapatite. FT-IR spectra obtained in the absence and presence of peptide were found to consistent with the formation of hydroxyapatite. Amide I (1653 cm^−1^), Amide II (1553 cm^−1^), Amide III (1261 cm^−1^) and also side-chain (1400–1500 cm^−1^) peaks were observed in the presence of peptide (line 2), indicating the presence of peptide in the reaction products.

The effect of the synthetic peptide on the behavior of spontaneous calcium phosphate precipitation was monitored through changes in pH as shown in **Figure 6**. Once nucleation took place, solution pH would reduce with time due to deprotonation of H_2_PO_4_^−^ and HPO_4_^2−^ ions associated with the formation of mineral precipitates. In the absence of peptide (control, line b), as previously reported (Margolis et al., 2014), a slow decrease in pH from 7.4 to 7.2 was seen during the first 15 min (**Figure 6**, line b), corresponding to the formation of spherical particles of ACP, as shown below. **Figure 7** shows TEM and SAED analyses of mineral phases formed during spontaneous mineralization experiments in the absence and presence of peptide (50 μg/mL). Mineral phase identification was based on the broad diffuse SAED ring pattern (**Figure 7A**, 15 min, insets) and the morphology of TEM observations (**Figure 7A**, 15 min), which exhibited spherical shapes that are consistent with the known morphology of ACP (Eanes, 2001; Zhang et al., 2016), similarly reported in other studies (Mahamid et al., 2008). Then, a rapid decrease in pH was observed from 7.2 to 6.5, in correspondence to the formation of plate-like particles. Spherical particles could no longer be found at 45 min (**Figure 7A**, 45 min), followed by a slow decrease in pH up to 24 h, at which point plate-like apatitic crystals were observed (**Figure 7A**, 24 h). SAED analyses showing the diffraction planes of (002), (112), and (004) (**Figure 7A**, 24 h, insets) were indexed as the hydroxylapatite crystallographic planes, suggesting it formed hydroxylapatite crystals, as confirmed by FT-IR (**Figure 8**, curve 1). However, in the presence of the synthetic peptide, TEM observations and SAED analyses revealed the formation of ACP particles at the first 15 min, 45 min and even after 2 h. These spherical particles were found to correspond with the PH change (**Figure 6**, line a). Furthermore, bundles of elongated ribbon-like crystals were observed after 24 h (**Figure 7B**, 24 h). The SAED pattern exhibited narrow diffraction arcs corresponding to (112) and (004) reflections (**Figure 7B**, 24 h, insets), indicating the formation of HA, as confirmed by FT-IR (**Figure 8**, curve 2). **Figure 8** shows FT-IR analyses of mineral phases formed after the 24 h in the absence of peptide (curve 1) and presence of peptide (curve 2). In the absence of peptide, FT-IR spectra were found to be consistent with the formation of the hydroxyapatite phase, with a absorbance peak at about 1100 and 1030 cm^−1^ arising from asymmetrical ν3 stretching vibrations of PO_4_^3−^ (Gadaleta et al., 1996; Smith, 1998; Wiedemann-Bidlack et al., 2011). In the presence of 50 μg/mL peptide (curve 2), asymmetrical ν3 stretching vibrations of PO_4_^3−^ were found at (1095 and 1029 cm^−1^), indicating the formation of hydroxyapatite crystals. At the peptide groups, Amide I (1653 cm^−1^), Amide II (1553 cm^−1^), Amide III (1261 cm^−1^) and also side-chain (1400–1500 cm^−1^) peaks were observed which suggested the presence of peptides in the reaction products.

## Discussion

Dental enamel is a hard natural bioceramic that protects the tooth. Its mechanical properties are associated with elongated and oriented prisms of hydroxyapatite (Moradian-Oldak, 2012). Dental enamel forms through protein-protein and protein–mineral interactions. As the enamel matures most of the proteins engaged in enamel biomineralization are subsequently degraded and eventually removed (Fincham et al., 1999) to allow final mineralization of intercrystalline spaces, resulting in mineral deposition in the form of hydroxyapatite crystals. Reconstructing enamel-like structures through organic–inorganic interactions is an emerging field in the material sciences and dentistry. To promote biomimetic mineralization, calcium phosphate paste that contains hydrogen peroxide (Yamagishi et al., 2005), amelogenin (Fan et al., 2009) and its functional domains such as leucine-rich amelogenin peptide (Le Norcy et al., 2011a), and a recent synthetic anionic oligopeptide amphiphile (Li et al., 2014) has been used. In this investigation, we designed and synthesized a novel biomimetic peptide that is composed of parts of the highly conserved N- (phosphorylated at S-16) and C- terminus of porcine amelogenin, as these two regions are critical for proper enamel formation (Paine and Snead, 1997; Paine et al., 2003).

We studied the remineralization efficacy of the synthetic peptide on bovine enamel *in vitro* using micro-CT. Bovine enamel was utilized as a substitute for human enamel in the present study, as there are only minute differences in chemical, physical characteristics and cariogenic challenge features between human and bovine substrates. Bovine enamel is obtained effortlessly and presents a relatively larger caries-free surface area with more uniform enamel thickness when compared with human enamel (Lippert and Juthani, 2015). In addition, flattening and polishing of bovine enamel surfaces can remove natural inter-sample variations on surface enamel to achieve standardization of specimens (Elkassas and Arafa, 2014). This operation would also expose a enamel surface with a higher porosity, which could leave the enamel more susceptible to acid attacks (Ranjitkar et al., 2009). X-ray micro-computed tomography (micro-CT) have gained increasing popularity for use in caries research and de/remineralization studies (Dong et al., 2014). As a non-destructive and quantitative tool, micro-CT can provide both two-dimensional (2D) and three-dimensional (3D) architectural information of samples. Moreover, it can measure the linear attenuation coefficient accurately, from which mineral content of the teeth can be determined. Therefore, micro-CT technology has been suggested as a reliable alternative to quantitatively characterize surface topography without contact. The micro-CT results showed that the mineral loss was significant and relatively stable after demineralization treatment among all the experimental groups (**Figure 4**), confirming the effectivity and stability of the DS. As expected, enamel lesions treated with fluoridated remineralization solution exhibited higher remineralization effect than all of the other treatment groups. Inclusion of this synthetic peptide in the remineralizing medium at concentrations of 25–50 μg/mL promoted greater remineralization of enamel lesions compared to remineralizing medium only. No significant difference was observed between the remineralizing medium treatment and the 12.5 μg/mL peptide treatment. A dose-dependent relationship was also observed among experimental groups treatment with peptides. Twelve point five microgram per milliliter peptide, 25 μg/mL peptide, 50 μg/mL peptide, and fluoridated remineralization solution resulted in a 22.51, 62.01, 119.75, and 187.64 increase in %R, respectively, compared with the remineralization solution as shown in **Table 1**. We found that there was more mineral gain in the lesion body when enamel was treated with peptide-containing remineralizing medium compared with the remineralizing medium-only group by analyzing the mineral density profiles of all groups. These results suggest that peptide treatment may influence the pattern of mineral deposition and allow ions to penetrate into deeper areas.

Artificial saliva was used as a remineralizing medium as previously reported (Ten and Duijsters, 1983; Chu et al., 2007). Its composition is close to the body’s physiological saliva and has been widely used for the study of dental caries remineralization. A study has reported that saliva is supersaturated with respect to the enamel mineral phases (Hay et al., 1982), thus providing a potential to support the mineral growth in the enamel caries. Our study confirms the ability of the artificial saliva to aid enamel remineralization and its ability to remineralize demineralized enamel crystals stems from its potential to supply bioavailable calcium and phosphate ions to the surfaces of enamel disks (Chu et al., 2007). However, the net remineralization produced by artificial saliva is a slow process and has a relatively small effect (Elkassas and Arafa, 2014), explaining why the control group showed the lowest values of remineralization among all groups. In this study, fluoride was incorporated in the remineralizing solution at a concentration of 2 ppm and the fluoridated remineralizing solution was supersaturated with respect to fluorapatite (Zhang et al., 2014). As expected, the highest remineralization potential was achieved after the fluoridated remineralizing solution treatment, which was consistent with results from the previous fluoridated treatment experiments (Ten, 2008; Zhang et al., 2014). The reason could be that this low concentration of fluoride can penetrate directly into fluoridated hydroxyapatite without the formation of spherical CaF2 particles, which may occur under high fluoride concentrations.

Previous studies have shown that full-length amelogenin and its proteolytically cleaved products tend to buffer the free calcium ion concentration through binding to calcium ions, as the negatively charged on the C-terminal regions and the phosphorylated serine residues on the N-terminal regions (Yamakoshi et al., 2001). Our findings have confirmed this ability by showing that the Ca^2+^/peptide interaction had a high affinity up to 9.914 × 10^4^M^−1^ and binding Ca^2+^ mainly through electrostatic, which may associate with the negatively charged on the C-terminal regions and the phosphorylated serine residues on the N-terminal regions. The values of the thermodynamic parameters changes, ΔH < 0 and ΔS > 0, indicating that the binding was mainly through electrostatic interaction and desolation (Zhou et al., 2016). As binding of calcium and phosphate ions, lowering the solution supersaturation and inhibiting the crystal growth. We also found that the presence of synthetic peptide can stablize ACP for more than 2 h until its transformation into elongated ribbon-like crystals at supersaturated solution (**Figure 7B**, 24 h). However, in the absence of peptide (control, **Figure 7A**), ACP was quickly transformed into randomly arranged crystals.

There is a defined surface layer during the early stages of enamel caries lesions and the demineralization mainly occurs in subsurface, so the repair should involve remineralization in the subsurface rather than just the superficial crystals (Eisenburger et al., 2001; Ganss et al., 2004). This implies that the precipitation of minerals on the surface cannot effectively recover the enamel lesions. In the remineralization results, a dose-dependent relationship was also observed among experimental groups treated with peptide. This is because different concentrations of the peptide stabilize ACP at different time scales similarly to native amelogenins (Kwak et al., 2014). The lower concentration stabilizes ACP quicker so calcium phosphorus do not have enough time to penetrate into the deeper caries lesions, and only form spontaneous deposition at the enamel surface. The higher concentration peptide allows calcium phosphorus enough time to penetrate into deeper layers. However, the 12.5 μg/mL peptide treatment did not show an improved remineralization effect versus the remineralizing medium only. One possible explanation could be that this concentration was still too low to stabilize ACP and the calcium phosphorus could not penetrate into deeper layers.

Previous studies have shown that utilizing casein phosphopeptides to stabilize ACP and deliver bioavailable calcium and phosphate ions into subsurface lesion can remineralize the enamel lesions (Cross et al., 2005; Cochrane et al., 2008; Reynolds et al., 2008). Our study has shown that our synthetic peptide have the ability to stabilize ACP similarly as casein phosphopeptides, therefore we hypothesize that the peptide may serve as a calcium ion carrier and a regulating factor. By binding Ca^2+^ and stabilizing ACP, it prevents spontaneous crystallization at supersaturated solutions, allowing enough time to transfer calcium and phosphorus from the remineralizing medium into the deeper layers of carious lesions through the micropore caused by demineralization. It also promotes calcium and phosphorus redeposition in deeper lesions; not only through spontaneous crystallization at the enamel surface, but by also guiding them to transform into ordered arrays of enamel-like crystals as the presence of the C-terminus. Briefly, when bovine enamel blocks were exposed to the peptide-containing remineralization medium, peptide may have attracted calcium and phosphorus from the artificial saliva in the form of ACP, and then penetrated into the deeper lesions through the micropore. It adheres to the hydroxyapatite of the enamel through the relatively hydrophilic C-terminus that contains the majority of the charged residues (Shaw et al., 2004), therefore peptide can promote calcium phosphate redeposition and guide ordered arrays of crystals at the deeper lesions. However, the 12.5 μg/mL peptide treatment did not show improved remineralization effect compared to the remineralizing medium only, perhaps due to: (1) further remineralization is simply not allowed to occur in the applied low peptide concentrations; (2) the *in vitro* remineralization-only models insufficiently simulate the real oral environment; and (3) limitations of the analysis methods used. So, further *in situ* studies should be conducted to exam the effects of 12.5 μg/mL synthetic peptide.

## Conclusion

This *in vitro* study preliminarily showed that specific concentrations of amelogenin-based synthetic peptide can increase the remineralization of incipient enamel lesions in a dose-dependent fashion: higher concentrations (>25 μg/mL) exhibited significantly greater remineralization. Mechanism of such effect is that: the peptide may act as a calcium ion carrier and also a regulating factor to guide ordered arrays of crystals form. It did also demonstrate that quantitative assessment using micro-CT was sensitive for detecting mineral density changes in bovine enamel. Our study provided the first step toward optimizing the peptide for testing *in vivo* and *in situ* to make this approach clinically viable. As the peptide has the potential to form ordered mineral crystals to restore enamel mechanical property, further studies should be conducted to verify its function of promoting remineralization of early enamel caries including animal model experiments.

## Author Contributions

JC, XF, HG, TZ, HZ, and QZ conceived and designed the experiments. XF and HG performed the experiments. XF, HG, and JC analyzed the data. JC, XF, HG, TZ, HZ, and QZ wrote the paper.

## Conflict of Interest Statement

The authors declare that the research was conducted in the absence of any commercial or financial relationships that could be construed as a potential conflict of interest.
